# The pandemic’s cruel aftermath: progressive decline in spay/neuter capacity

**DOI:** 10.3389/fvets.2025.1558235

**Published:** 2025-03-21

**Authors:** Simone D. Guerios, Gina Clemmer, Julie K. Levy

**Affiliations:** ^1^Department of Small Animal Clinical Science, College of Veterinary Medicine, University of Florida, Gainesville, FL, United States; ^2^Clinic HQ, Portland, OR, United States; ^3^Shelter Medicine Program, College of Veterinary Medicine, University of Florida, Gainesville, FL, United States

**Keywords:** SARS-CoV-2, COVID, pet overpopulation, castration, sterilization, shelter medicine, ovariohysterectomy

## Abstract

The COVID-19 pandemic lockdown in early 2020 resulted in a temporary suspension of elective spay and neuter procedures in many low-cost spay/neuter clinics. In our previous study, we projected a deficit of 2.7 million surgeries performed in high-quality high-volume spay-neuter (HQHVSN) clinics as a result of the shutdown and subsequent inability to recover to pre-pandemic productivity by the end of 2021. The purpose of this follow-up study was to determine whether the clinics subsequently recovered and caught up with the previously delayed procedures. Spay-neuter data were collected from 212 HQHVSN clinics from January 2019 through June 2023. The clinics collectively performed 1,217,240 spay/neuter surgeries in the pre-COVID baseline year of 2019. The pandemic triggered a reduction of 13% in 2020, 3% in 2021, 6% in 2022, and 1% in the first half of 2023. Analysis of patient data from the same clinics in our previous report revealed that instead of rebounding to pre-pandemic surgery capacity, they performed even fewer surgeries per quarter in the 18-month follow-up period than they did in 2021. If similar trends occurred in the estimated 3,000 spay-neuter clinics across the United States, the deficit in spay-neuter surgeries is estimated to have risen to 3.7 million surgeries, not including the compounding effect of those intact animals producing litters of their own. The continued decline in low-cost spay-neuter year over year impedes access to basic preventive pet healthcare and threatens to undermine decades of progress in controlling pet overpopulation.

## 1 Introduction

Spay/neuter surgeries, mostly conducted by low-cost clinics, have contributed to a significant reduction in unwanted litters and, consequently, a reduction in animal euthanasia in shelters over the past 50 years (1). The COVID-19-related epidemiological situation has affected veterinary services in the United States (2), including a significant reduction in the number of spay-neuter surgeries. In our previous study, we reported a 13% reduction in spay/neuter surgeries performed during the pandemic year of 2020 and a 3% reduction in the number of surgeries performed in 2021 (3). Three years after the initial pandemic shutdowns, spay-neuter clinics continue to struggle to meet the demand for services. Coupled with the current shortage of veterinarians and staff that further threatens spay/neuter recovery (4, 5), these conditions are conducive to an increase in unwanted litters, overcrowding in animal shelters, and an increase in euthanasia rates. This follow-up study aimed to determine whether spay-neuter clinics recovered the number of surgeries performed in the 18-month post-pandemic period following our previous report.

## 2 Materials and methods

A retrospective study was conducted using dog and cat spay-neuter data collected by Clinic HQ (Clinic HQ Inc., Portland, OR), a cloud-based clinic management software program designed for practices focusing on spay-neuter and preventive healthcare services. Spay/neuter surgery numbers from January 2019 through June 2023 were recorded. A unique identification number was assigned to each clinic, and the information was collected and stored electronically. No identifiable client, patient, or clinic information was stored with the data available for the study.

Quarterly trends were compared using data from January 2019 through June 2023. The year 2019 was considered the pre-pandemic baseline, 2020–2021 was considered the pandemic period, and 2022–2023 was considered the post-pandemic period.

## 3 Results

Of the original 212 HQHVSN clinics tracked since 2019, five underwent mergers, and two discontinued using Clinic HQ software. As of June 2023, only 161 clinics (76%) had remained open for surgery throughout the 4.5-year study period. A total of 28 clinics (13%) temporarily suspended surgery, 24 of which reopened within 4 months, while four others paused surgery for 9 to 38 months. Twelve clinics (6%) permanently closed, and six clinics (3%) stopped performing surgeries and converted to wellness services only.

**Figure 1:**
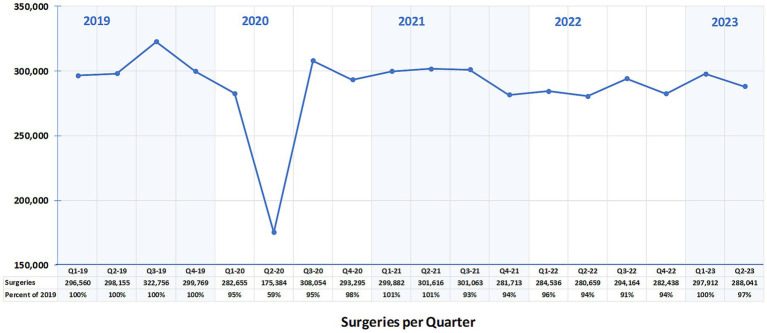
Number of cats and dogs spayed or neutered per quarter at 212 spay-neuter clinics in the US during baseline year 2019, COVID-19 pandemic years 2020–2021, and post-pandemic years 2022–2023.

The total number of spay-neuter surgeries for the baseline year 2019 was 1,217,240. Compared to the baseline year, a reduction of 13% (1,059,388) in 2020, 3% (1,184,274) in 2021, 6% (1,141,797) in 2022, and 1% (585,953) in the first two quarters of 2023 was observed (Figure 1).

**Figure 2:**
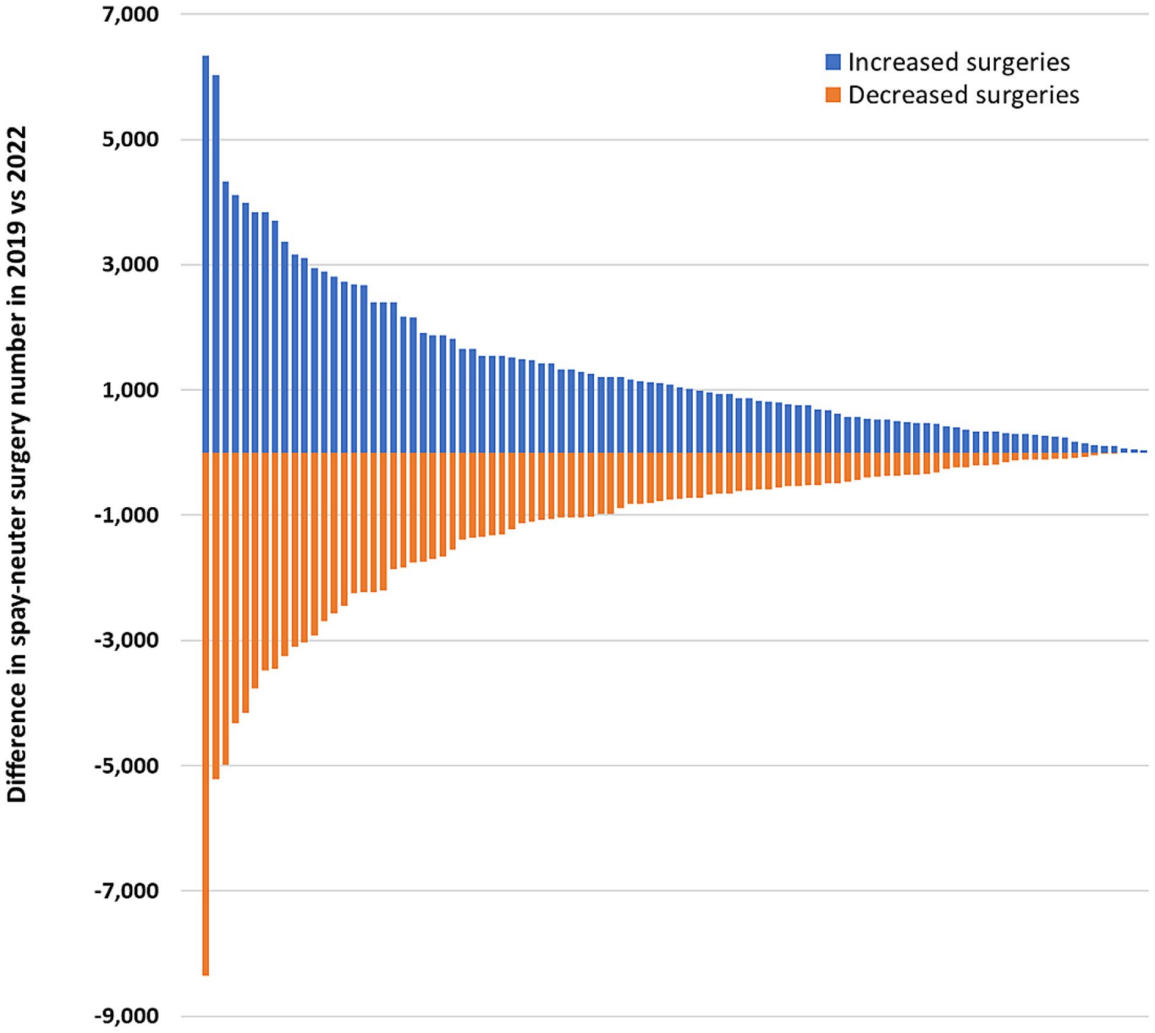
Increased (96 clinics) or decreased (93 clinics) number of spay-neuter surgeries performed in 2022 compared to the number performed in the baseline year of 2019 at each of 189 clinics that performed surgeries each year. In addition to these clinics, 12 clinics (6%) permanently closed, and six clinics (3%) stopped performing surgeries and converted to wellness services only.

Of 189 clinics that conducted surgeries in both 2019 and 2022, 96 clinics together increased their surgeries by 135,601 in 2022, while 93 clinics performed 118,423 fewer surgeries (Figure 2), resulting in a net increase of 17,178 surgeries overall. However, the closure or discontinuation of surgery at 18 clinics led to an overall decrease of 70,943 surgeries in 2022. Data for surgeries in 2023 was available only for the first two quarters, and during that period, the numbers slightly lagged behind those of the first half of 2019.

In total, 261,763 fewer surgeries were performed by the 212 studied clinics during the 42 months from January 2020 through June 2023 than would have been expected had 2019 levels been maintained. If a similar pattern was experienced by all 3,000 estimated spay-neuter clinics in the US, it would suggest a deficit of more than 3.7 million spay-neuter surgeries have accumulated by mid-2023.

## 4 Discussion

The COVID-19 pandemic resulted in a substantial decrease in spay-neuter surgeries. Our previous study projected a deficit of 2.7 million surgeries performed in HQHVSN clinics as a result of the shutdown and subsequent inability to recover to pre-pandemic productivity by the end of 2021 (3). This follow-up study of the same clinics revealed an ongoing decrease in the number of surgeries during the post-pandemic period of 2022 through mid-2023, demonstrating that the spay/neuter clinics included in the study have yet to recover their pre-pandemic capacity.

The high level of spay-neuter achieved over the past five decades is the most significant factor in reducing pet overpopulation and has increased access to veterinary care (6). HQHVSN clinics have been offering low-cost spay and neuter surgeries and wellness care, including vaccinations and parasite preventives, as an affordable alternative path for families who cannot afford comprehensive veterinary care (2, 7). Additionally, they also help shelters and pet rescue organizations sterilize the animals in their care, which increases their adoption appeal and reduces shelter crowding and length of stay.

Decreased low-cost services provided by HQHVSN clinics leave pet owners with the option of higher-priced care at traditional veterinary clinics or forgoing care altogether. The price of veterinary care is increasing faster than inflation overall, further exacerbating barriers to access (8). As the total number of veterinary visits decreases year over year, fewer pets are receiving necessary veterinary attention, which contributes to emotional distress when these pets are considered to be part of the family (9, 10). Veterinary and animal shelter staff also suffer psychological distress when pets are left without medical care, undergo economic euthanasia, or are relinquished to shelters (5, 10–12). Veterinary care costs are mentioned as a leading barrier to the acquisition of a new pet, particularly in lower-income families, which are also more likely to relinquish a pet to a shelter (13). Together, these financial limitations on the human-animal bond also contribute to overcrowding of animal shelters, longer lengths of stay for shelter pets, and higher euthanasia rates for homeless pets (13, 14).

Limitations of this study included lack of data from HQHVSN clinics using other software and data from private veterinary practices. However, the pandemic and subsequent national veterinary workforce shortage likely had negative impacts on elective surgery numbers across all sectors of the veterinary industry. Another limitation is the fact that there is only a single year of pre-pandemic baseline data. A longer multi-year period would help capture fluctuations that may have occurred in the number of spay-neuter surgeries before the pandemic year of 2020. It would also permit enumeration of broad trends, such as the generally increasing numbers of surgeries performed in HQHVSN clinics year over year prior to the pandemic, which would have allowed for our analysis to account for the deficit in unrealized clinic productivity growth in addition to a static comparison to surgeries completed in 2019.

In conclusion, our study suggests that the ongoing decline in low-cost spay-neuter services since the pandemic has limited access to basic preventative healthcare for pets, threatening to reverse decades of progress in managing pet overpopulation and jeopardizing the human-animal bond in the most vulnerable families.

## Data Availability

The raw data supporting the conclusions of this article will be made available by the authors, without undue reservation.
